# Digitally Delivered, Group-Based Exercise Interventions for Older Adults: Scoping Review

**DOI:** 10.2196/73578

**Published:** 2025-09-03

**Authors:** David Wing, Jeanne F Nichols, Maira Tristao Parra, Hava Shoshana Barkai, Ryan J Moran

**Affiliations:** 1 Exercise and Physical Activity Resource Center Herbert Wertheim School of Public Health and Human Longevity Science University of California, San Diego San Diego, CA United States; 2 International Consulting Associates Arlington, VA United States; 3 General Internal Medicine School of Medicine University of California, San Diego San Diego, CA United States

**Keywords:** online instruction, older adults, functional fitness, physical training, strength, balance, fall prevention, videoconferencing, Zoom, Skype

## Abstract

**Background:**

Falls and fractures are the leading cause of unintentional injury among older adults, resulting in increased mortality and morbidity, as well as reduced physical function and quality of life. In-person exercise programs aimed at improving strength, balance, and postural control have demonstrated benefits for physical function, quality of life, and fall risk reduction among older adults. Technology-driven approaches can further enhance the accessibility of exercise programs. In particular, digitally delivered programs offer the opportunity to balance risks and benefits while promoting engagement and potentially improving physical function.

**Objective:**

The overall aim of this review was to summarize the growing body of research on the efficacy, usability, and safety of these programs in older adults.

**Methods:**

MEDLINE via PubMed, the Cochrane Controlled Register of Trials (CENTRAL), and Embase databases were searched for this review. The initial search was conducted in November 2022 and updated in July 2024. Randomized controlled trials, nonrandomized trials, and single-arm pilot studies of at least 6 weeks’ duration reporting digitally delivered exercise for presumptively healthy older adults, taught in real-time (not prerecorded) by a qualified instructor, were included. Interventions targeting specific clinical subpopulations (eg, cardiac rehabilitation, Parkinson disease, chronic obstructive pulmonary disease) were excluded, although common age-related conditions such as hypertension, diabetes, and osteoporosis were included. The review was preregistered via INPLASY (registration number 3773).

**Results:**

A total of 4242 studies were screened by title and abstract, with 76 progressing to full-text review. Of these, 23 (30%) met all inclusion criteria, comprising 6 pilot single-arm studies, 5 nonrandomized trials, and 12 randomized trials. Interventions ranged from 6 to 24 weeks, with most lasting 8-12 weeks, and class participation typically occurred 2-3 days per week. Class sizes ranged from as few as 4 to more than 30 participants. Instructor experience varied and included licensed professionals, such as physical therapists, kinesiologists, and certified trainers, as well as laypeople specifically trained for the intervention. A total of 18 out of 23 (78%) studies reported physical outcomes, including balance, strength, and functional measures. Fourteen of these studies reported clinically meaningful improvements following the intervention, most commonly in strength and balance, measured by the 30-second chair stand test and the timed up and go; 20 studies (87%) reported 1 or more observations regarding safety or program usability. Among the studies that provided data on adverse events, most were conducted without injuries or reported only minor injuries. More than 60% of the authors (15/23, 65%) noted in their conclusion statements that participant acceptance of the digital delivery format was high.

**Conclusions:**

Overall, these findings demonstrate partial effectiveness in improving physical function related to fall prevention among older adults. Additionally, high attendance, participant enjoyment, and safety highlight the utility of digitally delivered exercise programs for older adults taught in real time.

**Trial Registration:**

International Platform of Registered Systematic Review and Meta-analysis Protocols (INPLASY) INPLASY202280097; https://inplasy.com/inplasy-2022-8-0097/

## Introduction

### Background

Falls represent a significant cause of preventable injury, contributing to premature mortality and morbidity. Fall-related injuries are the major cause of accidental death and disability among older adults, with approximately one-quarter of community-residing men and women over 65 years of age, and almost half of those over age 80, falling annually [1]. Alarmingly, the rate of fall-related mortality increased by more than 30% between 2007 and 2016 [2]. In the last decade, digitally delivered basic medical care has increased substantially [3,4] and has been adopted as a key modality of care by many medical centers. While most older adults appear to prefer in-person visits for their medical care, many believe that telemedicine is useful and should continue to be available, particularly for more routine appointments [5,6], reflecting an increased awareness of the importance and acceptance of this modality. In addition to delivering medical care, there are opportunities for providing preventive care through digital technologies, including fall-risk screening with follow-up participation in evidence-based exercise programs. However, given that many older adults have expressed difficulty with technology [5,6], improving our understanding of how technology-driven approaches can be organized to support older adults is critical. These methods may further enhance telemedicine’s ability to provide care, including real-time interventions to reduce fall risk safely and effectively in older adults.

### Types of Programs That Have Proven Efficacious for Improving Physical Function and Preventing Falls

While fall risk is multifactorial, exercise targeting a combination of dynamic balance and functional strength appears to be the most efficacious in reducing fall incidence and improving physical function in adults aged 60 years and older [1]. In particular, structured exercise programs such as Tai Chi [7] and the Otago program [8] have been effective in reducing both fall risk and the rate of falls in older adults. While successful programs differ somewhat in their areas of emphasis and rate of progression, the most effective programs typically emphasize progressive balance training and lower limb strengthening [1]. Indeed, in a comprehensive systematic review and meta-analysis published in 2020, Sherrington et al [9] reported a 34% reduction in fall rate among older adults participating in more than 3 hours per week of total exercise that incorporated systematic balance and strength training. Although specific analyses incorporating dose-response based on attendance are relatively sparse, it appears that interventions prescribing a greater number of practice days (either directly instructed or at home) yield greater improvements in both fall risk and number of falls [10]. More specific descriptions of some exercise interventions identified as effective for reducing fall risk can be found in the Centers for Disease Control and Prevention Compendium of Effective Fall Interventions [1].

### Digitally Delivered Fall Prevention Programs

Digital delivery of fall prevention programs may provide an opportunity to improve dissemination and help balance risks and benefits. Such programs may also serve communities lacking appropriate facilities or trained personnel for in-person programs. Similarly, digital programming can reduce barriers for individuals who are unlikely or unable to attend in-person classes due to disease [11], disability [11,12], or geographic remoteness [13,14].

While hundreds of exercise videos are available on the internet—many of which are likely appropriate for older adults—most feature an instructor leading a routine alone in front of the camera, or with a small group of participants in a studio setting. Given the recorded nature of this format, feedback from the instructor is not tailored to the individual performing the exercises and is typically limited to group “cheerleading” in the form of generalized encouragement or nonspecific instruction regarding safety, technique, or progression. Even for classes taught live but with a single instructor, it is likely very difficult for the instructor to provide individualized and targeted feedback to a specific participant who clearly needs form correction in real time. Given the evidence suggesting that video-based instruction has limited (ie, not statistically significant) impact on fear of falling [15] and rate of falls [16], that engagement in a video-based exercise intervention decreases rapidly [16], and that improper technique is a key contributor to exercise-based injury [17], classes that are either video-based or involve large groups taught by a single instructor likely have limited utility.

### Significance and Aims of This Review

This scoping review of real-time, virtually delivered, instructor-led exercise trials aims to summarize the growing body of research addressing the efficacy, usability, and safety of this type of program for older adults. In particular, we aim to summarize instruction methodologies and intervention outcomes, as well as identify gaps within the existing literature.

## Methods

### Overview

Following the standards established in 2018 to guide scoping reviews [18], we defined the review objectives, developed a protocol for data extraction and inclusion, and conducted independent, blinded data analysis by 2 investigators, followed by discussion among investigators to resolve conflicting interpretations. These processes are detailed more thoroughly below. The review was preregistered with INPLASY (registration number 3773).

### Search Strategy

A systematic search of MEDLINE via PubMed, the Cochrane Controlled Register of Trials (CENTRAL), and Embase was conducted for peer-reviewed (nonpreprint) publications available in English through July 2024, with no limit on start date. Combined, these 3 databases maximize international reach, covering countries in North America, much of Latin America, and most of Europe, Africa, and Asia. See Multimedia Appendix 1 for the search terms used. Additionally, the bibliographies of systematic reviews identified by the primary search strategy were searched to identify potentially eligible studies.

### Study Inclusion Criteria

Interventional study designs, including randomized controlled trials, nonrandomized trials, and single-arm pilot studies, were included in the search. Interventions were eligible only if they were digitally delivered (eg, via Zoom or another similar platform), provided instruction in real time to a group of more than 3 individuals, and lasted a minimum of 6 weeks. We did not require assessments to be digital; therefore, many interventions that included physical measurement conducted these assessments in person. Classes had to include primarily balance and strength training, or other modes of exercise focused on fall prevention, such as Tai Chi, Yoga, or Pilates. This criterion was based on the understanding that, while not necessarily recommended as first-line interventions to reduce fall risk [19], these modalities do appear to contribute to improvements in balance and strength [20,21].

### Participant Inclusion/Exclusion Criteria

As a first step in evaluating the scope of group-based, digitally delivered exercise programming for geriatric populations, we chose to focus on apparently healthy older adults. Substantial details regarding inclusion and exclusion criteria are provided in Textbox 1.

**Inclusion and exclusion criteria.**

1. Inclusion criteria
  - Interventional study design.
  - Exercise delivered digitally in real time by live instructors.
  - Participant group size greater than 3 individuals, with intervention duration of at least 6 weeks.
  - Intervention focused primarily on balance and muscular strength.
  - Generally healthy older adults living independently or semi-independently.
2. Exclusion criteria
  - No intervention or purely observational study.
  - Exercise delivered via video recording or other asynchronous methods.
  - Intervention delivered in-person (unless serving as a control group).
  - Individualized or short-term interventions.
  - Interventions focusing exclusively on aerobic fitness.
  - Participants with known cardiac, endocrine, or neuropsychological diseases, or severe cognitive impairment.

Given the older adult population, many of the studies included participants with minor or controlled conditions, such as hypertension, sarcopenia, and mild cognitive impairment. However, in this context, the apparently healthy sample excludes patient populations with severe cardiac, endocrine, or neuropsychological disease, or severe cognitive impairment. These individuals were excluded due to the likelihood of needing additional support in the form of specialized facilities, safety equipment, or clinical training, which would make remote delivery increasingly challenging, particularly in a group-based setting. Additional requirements were being 60 years of age or older; living independently or semi-independently (eg, residing in an assisted living facility without dependency on any care other than meals); being ambulatory without any assistive device; and being able to participate in exercise according to the primary study author’s criteria.

Specific subpopulations excluded from selection were patients with cardiac problems or those with diagnosed cardiovascular disease, individuals with uncontrolled hypertension, diagnosed neuromuscular or musculoskeletal disorders (including Parkinson disease), other conditions requiring ongoing medical treatment, and individuals diagnosed with severe cognitive impairment or deemed unable to follow the instructions of an exercise leader.

## Results

**Figure 1:**
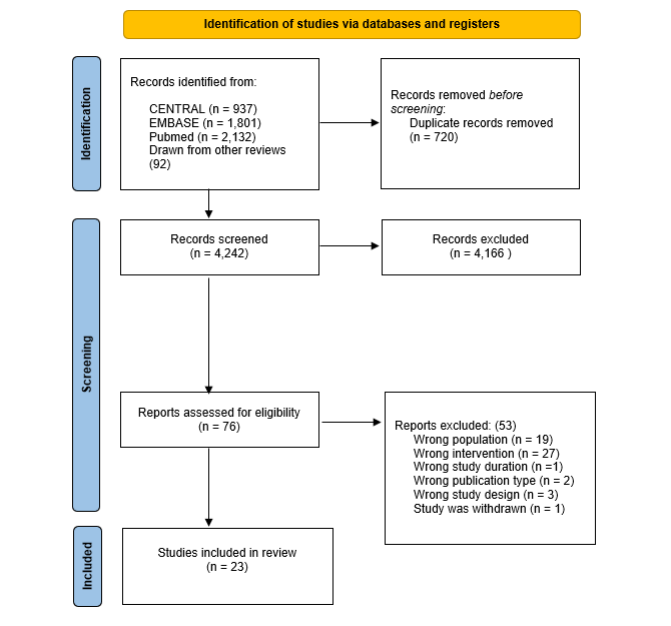
Identification of studies via databases and registers.

As indicated in Multimedia Appendix 2, we followed the PRISMA-ScR (Preferred Reporting Items for Systematic reviews and Meta-Analyses extension for Scoping Reviews) checklist. We identified 4870 records, and after removing duplicates, 4242 were screened independently by 3 reviewers for titles and abstracts that appeared to match the inclusion/exclusion criteria described above. When 2 or more reviewers agreed on an apparent match, records were advanced to the next stage. Reasons for exclusion during this phase were not systematically recorded. A total of 76 records were retrieved and reviewed in full text, and 23 studies fulfilled all criteria and were included in this review. Two investigators, blinded to each other, extracted data on study elements related to the intervention methods and study results. The headers of the extracted data are provided in Multimedia Appendix 3. Following this independent review, the 2 investigators jointly checked the data extractions for omissions and possible discrepancies, with support from the third investigator. This data flow is shown graphically in Figure 1.

Details on key demographics of the study samples, interventional methods, outcome measures, and results of the 23 reviewed studies are presented in Tables 1 and 2—12 of the 23 studies were nonrandomized trials (n=5) or single-group (n=7) observational studies reporting pre/postexercise intervention results; the remaining 11 were randomized controlled trials. The studies were conducted across 14 countries: the United States (n=7), Canada (n=3), Cyprus (n=2), and 1 each from Australia, Brazil, England, France, Germany, Greece, Israel, Malaysia, Mexico, South Korea, and Turkey.

**Table 1:** Sample and intervention characteristics.

Author and citation			Key sample demographics		Characteristics of intervention and control	
**Randomized controlled clinical trials**						
	Ing et al [22]	Country: Malaysia Sample size: 52 (intervention, n=26; control, n=26) Age (years), mean (SD): 66.5 (5.2) Female, n (%): 44 (85)		Duration: 24 weeks Characteristics: 2/week × 75-90 minutes; focused on strength and balance via Zoom; supervised by a physical therapist with 10 years’ experience; and class size not given. Control characteristics: One session of virtual fall prevention education.		
	Maranhão et al [23]	Country: Brazil Sample size: 38 (intervention, n=18; control, n=20) Age (years), mean (SD): 68 (7) Female, n (%): 31 (82)		Duration: 12 weeks Characteristics: 3/week × 25 minutes; focused on 7 weight-bearing exercises; direct virtual instruction delivered by certified professional; class size not given. Control characteristics: Identical prescription with no direct instruction.		
	Blioumpa et al [24]^a^	Country: Greece Sample size: 30 (intervention, n=15; control, n=15) Age (years), mean (SD): 60.3 (9.3) Female, n (%): 7 (32)		Duration: 6 weeks Characteristics: 3/week × 60 minutes (20-minute aerobics, 20-minute strength, 20-minute warm/cool) via Skype; supervised by a physical therapist; class size up to 4 participants. Control characteristics: Classes providing health-related educational content.		
	Terkes et al [25]	Country: Turkey Sample size: 70 (intervention, n=35; control, n=35) Age (years), mean (SD): 69 (6.6) Female, n (%): 31 (44)		Duration: 6 weeks Characteristics: 3/week × 60 minutes; focused on mobility, aerobics, strength, balance, flexibility via Zoom; delivered by a physical therapist; class size not given. Control characteristics: Same program with no direct supervision.		
	Okpara et al [26]^b^	Country: Canada Sample size: 70 (intervention, n=35; control, n=35); 67 completed baseline measures (socialization, n=32; multimodal, n=35) Age (years), mean (SD): 77 (6.5) Female, n (%): 52 (72.2)		Duration: 12 weeks Characteristics: 2/week × 60 minutes/session; focused on aerobic capacity, strength, and balance; delivered via Zoom, led by 1 physical therapist; class size 5. Control characteristics: One phone call/week to provide social interaction.		
	Granet et al [27]	Country: Canada Sample size: 83 (intervention, n=38; control, n=45) Age (years), mean (SD): 70.2 (5.2) Female, n (%): 68 (82)		Duration: 12 weeks Characteristics: 3/week × 55 minutes/session; focused on muscle function, flexibility, and cardiovascular capacity; delivered by a kinesiologist using Zoom; class size 11-14, capability matched. Control characteristics: Identical program provided by video.		
	Langeard et al [28]	Country: France Sample size: 43 (digital, n=15; face-to-face, n=15; control, n=13) Age (years), mean (SD): 73 (4) Female, n (%): 27 (66)		Duration: 16 weeks Characteristics: 2/week × 60 minutes/session via videoconferencing; focused on muscle function, functional activities, and aerobic capacity; led by a single trainer with adaptive physical exercise experience; class size 4. Control characteristics: Identical program done face-to-face; the no-training control group received no exercise.		
	Li et al [29]^c^	Country: The United States Sample size: 69 (enhanced Tai Ji Quan, n=23; standard Tai Ji Quan, n=22; control, n=24) Age (years), mean (SD): 74.6 (5.6) Female, n (%): 39 (56.5)		Duration: 16 weeks Characteristics: Standard Tai Ji Quan (2/week × 60 minutes/session); Tai Ji Quan (balance and coordination) via Zoom; led by instructors with 10+ years of experience; class size 4-6. Enhanced Tai Ji Quan: Tai Ji Quan with cognitive exercises. Control characteristics: Stretching (flexibility).		
	Yerlikaya et al [30]^d^	Country: Cyprus Sample size: 50 (interactive telehealth home exercise, n=18; nonsupervised telehealth home exercise, n=16; control, n=16) Age (years), mean (SD): 72.6 (6.9) Female, n (%): 35 (70)		Duration: 8 weeks Characteristics: interactive telehealth home exercise—3/week × 40 minutes/session remotely; focused on strength and balance; supervised by physical therapist; nonsupervised telehealth home exercise—8 weeks, 3/week × 40 minutes/session, only first session supervised; class sizes not given. Control characteristics: No change to behavior.		
	Li et al [31]^e^	Country: The United States Sample size: Tai Ji Quan, n=15; stretching, n=15 Age (years), mean (SD): 76.2 (6.2) Female, n (%): 21 (70)		Duration: 24 weeks Characteristics: 2/week × 60 minutes; Moving for Better Balance Fall Prevention Program; delivered via Zoom; class size 4-8. Control characteristics: Stretching exercises.		
	Tomita et al [32]	Country: The United States Sample size: 51 (intervention, n=25; control, n=26) Age (years), mean (SD): 73.2 (7.8) Female, n (%): 45 (88)		Duration: 24 weeks Characteristics: 3/week × 25-40 minutes; focused on strength of lower limbs and balance; delivered by a member of research staff with unknown qualifications; class size 6-7. Control characteristics: Minimal contact but encouraged to walk.		
**Interventional nonrandomized trials**						
	Canton-Martínez et al [33]	Country: Mexico Sample size: 44 (exercise without comorbidities, n=11; exercise with comorbidities, n=9; healthy control, n=15; comorbidity control, n=9) Age (years), mean (SD): not reported Female, n (%): not reported		Duration: 12 weeks Characteristics: 3 days/week × 60 minutes, virtually; supervised by trained staff; class size 20. Control characteristics: No change to lifestyle.		
	Pitre et al [34]	Country: Canada Sample size: 159 (analyzed, not including drops) (intervention, n=75; control, n=84) Age (years), mean (SD): 67 (7) Female, n (%): 136 (86)		Duration: 12 weeks Characteristics: 2/week × 60 minutes; focused on aerobics, balance, flexibility, and strength via Microsoft Teams; class size 20-25; instructed by peer leaders with specialized training. Control characteristics: Same intervention provided face-to-face; instructed by peer leaders with specialized training.		
	Aksay [35]	Country: Germany/Turkey Sample size: 357 (intervention, n=162; control, n=195) Age (years), mean (SD): 69 (7) Female, n (%): 231 (65)		Duration: 20 weeks Characteristics: 1/week × 60 minutes; focused on strength, endurance, balance, and flexibility using body weight; class size 8-15; intervention provided by a licensed instructor. Control characteristics: Maintain normal activities.		
	Buckinx et al [36]	Country: Canada Sample size: 44 (intervention, n=11; control, n=33) Age (years), mean (SD): 79.3 (6.2) Female, n (%): 30 (68)		Duration: 12 weeks Characteristics: 3/week × 60 minutes; focused on strength, balance, and light aerobics; taught via Zoom; supervised by a kinesiologist; class size not given. Control characteristics: Same prescription in print.		
	VanRavenstein et al [37]^f^	Country: The United States Sample size: 12 (intervention, n=6; control, n=6) Age (years), mean (SD): 72.3 (not reported) Female, n (%): 11 (91.6)		Duration: 12 weeks Characteristics: 2/week × 30 minutes; focused on balance and strength; delivered via an unspecified telehealth tool; trained instructor; drawn from the Otago program; class size 6. Control characteristics: Same program delivered face-to-face.		
**Interventional single-arm trials**						
	Lim et al [38]^g^	Country: England Sample size: 30 Age (years), mean (SD): 77.3 (5.5) Female, n (%): 27 (90)		Duration: 26 weeks Characteristics: 1/week × 30 minutes; seated and focused on strength and flexibility; mostly digital (a few clubs returned to face-to-face); led by trained volunteers supervised by an exercise instructor; class size not given. Control characteristics: Not reported.		
	da Silva et al [39]^h^	Country: Brazil Sample size: 89 original; completers (>70% attendance) measured and included in analyses, n=20 Age (years), mean (SD): 70.5 (4.4) Female, n (%): 18 (90)		Duration: 39 weeks Characteristics: 3/week × 60 minutes; focused on balance, strength, aerobic fitness, and flexibility; delivered via Facebook; led by 1 instructor and 1 assistant; class size not given. Control characteristics: Not reported.		
	Kirwan et al [40]^i^	Country: Australia Sample size: 171 Age (years), mean (SD): 71.5 (5.6) Female, n (%): 117 (68)		Duration: 8 weeks Characteristics: 2/week sessions (time per session not reported); focused on aerobics, strength, flexibility, and balance via Zoom; led by an accredited exercise specialist; class size 6. Control characteristics: Not reported.		
	Lee et al [41]^j^	Country: The United States Sample size: total, n=83; answered survey/analyzed, n=31 Age (years), mean (SD): answered survey, 72.3 (5.6); did not answer survey, 69.4 (6.2) Female, n (%): answered survey, 25 (80.6); did not answer survey, 43 (82.7)		Duration: 8 weeks Characteristics: Frequency of sessions and duration of classes not given; delivered via Zoom; led by 1 instructor and 1 assistant; class size 32. Control characteristics: Not reported.		
	Bagkur et al [42]	Country: Cyprus Sample size: 23 Age (years), mean (SD): 73.0 (5.13) Female, n (%): 15 (65)		Duration: 8 weeks Characteristics: 3/week × 40 minutes per session; focused on aerobic capacity, strength, balance, flexibility, and coordination; delivered via multiple teleconferencing apps; led by a physical therapist; class size not given. Control characteristics: Not reported.		
	Schwartz et al [43]^k^	Country: Israel Sample size: 31 Age (years), mean (SD): 71.5 (4) Female, n (%): 20 (64.5)		Duration: 8 weeks Characteristics: 2/week × 45 minutes per session; focused on aerobic and strength; delivered via Zoom by experienced instructors; class size 15. Control characteristics: Not reported.		
	Wu and Keyes [44]^l^	Country: The United States Sample size: 17 Age (years), mean (SD): 81 (8) Female, n (%): 13 (76.5)		Duration: 15 weeks Characteristics: 3/week × 60 minutes per session; focused on flexibility, strength, balance, and coordination; delivered via internet-based videoconferencing; led by an exercise instructor; class size 6-11. Control characteristics: Not reported.		

^a^Individualized heart rate–monitored prescription.

^b^Clinical frailty score 4-6 (mild to moderate); tool used not relevant.

^c^All participants had mild cognitive impairment; mobility—independent living.

^d^The control group was substantially older.

^e^30% poor or very poor self-reported health status; 50% with self-reported chronic conditions.

^f^100% Black race.

^g^Moderate level of comorbidity (Charlson Index=4).

^h^Over 50% had hypertension; 50% had osteoarthritis.

^i^All participants were diagnosed with type 2 diabetes mellitus.

^j^Less than 40% responded to the final survey; about 60% had arthritis; and over 35% had hypertension.

^k^Independent and ambulatory.

^l^Three participants depended on a walker.

**Table 2:** Measures and outcomes of reviewed studies.^a^

Study and its design			Measures		Outcomes
**Randomized controlled clinical trials**					
	Ing et al [22]	Physical: timed up and go, single-leg stand, hand grip, and time to complete 5 chair stands Survey: Baecke Physical Activity Score, Geriatric Depression Scale (Depression), EQ-5D (Quality of Life), and Falls Efficacy Scale-International (Fear of Falling) Usability: attrition, attendance, engagement, semistructured interview, and adverse events		Physical: significant intervention effects on timed up and go, single-leg stand, hand grip, and time to complete 5 chair stands Survey: post improved vs pre for Falls Efficacy Scale-International and EQ-5D, no group × time interaction Usability: 96% completed, 81% attendance, and 8/10 participant engagement during fidelity checks; interviews (n=6) indicated population had limited technology exposure, specific instruction regarding exercise was helpful, class length and cadence were good, and participants preferred videos with supervised completion versus live demonstration Adverse events/outcomes: 9 musculoskeletal pain events (believed unrelated)	
	Maranhão et al [23]	Physical: 30-second chair stands with Derived Muscular Power, time to complete 5 chair stands, and rise from floor to stand Usability: adherence Cognitive (additional): trails making A and B, Stroop Test, and Semantic Verbal Fluency of Animals		Physical: no significant within-group changes Usability: adherence—virtually supervised group 60% and minimally supervised group 83% Adverse events/outcomes: not reported	
	Blioumpa et al [24]	Physical: glycemic control (glycated hemoglobin [HbA1c]), 6-meter walk time, hand grip, 30-second chair stands, anthropometry, and BMI Survey: International Physical Activity Questionnaire (physical activity) and 36-item Short Form Survey (quality of life) Usability: attrition		Physical: glycated hemoglobin (HbA1c) decreased in the intervention group, not in the control group, group × time interaction in 6-meter walk time and hand grip with post>pre for the intervention group and post=pre for the control group, 30-second chair stands improved in the intervention group, not the control group, and decreased weight and BMI in the intervention group with no change in the control group. Survey: mental health and general health subscales significantly increased in the intervention group, but no change in the control group Usability: 73% completed (4 drops per group) and no serious exercise-related adverse events in the intervention group	
	Terkes et al [25]	Physical: blood glucose and BMI Survey: The Brief Resilience Scale and Control, Autonomy, and Self-Realization and Pleasure-19 (quality of life) Usability: not measured		Physical: post<pre for intervention for blood glucose without group × time interaction, group × time interaction favoring intervention, and post<pre in intervention for BMI Survey: the Brief Resilience Scale showed group × time interaction favoring intervention with both intervention and control post<pre, quality of life (total and perceived autonomy and satisfaction) showed group × time interaction favoring intervention with both intervention and control post>pre, quality of life (perceived disability) intervention>control at post (equal at pre) and both intervention and control post>pre with group × time interaction favoring intervention Usability: adverse events not reported	
	Okpara et al [26]	Physical: adverse events and time to complete 5 chair stands Survey: 21-item Depression, Anxiety and Stress Scale, Frailty Index, and EQ-5D-5L index Usability: attendance (percentage of classes attended) and study satisfaction (study-specific questionnaire)		Physical: no statistically significant difference between the groups in either the intention-to-treat and the per-protocol analyses for time to complete 5 chair stands. Survey: no statistically significant difference between the groups in either the intention-to-treat and the per-protocol analyses for depression, anxiety, and stress. Usability: attendance=81% of classes attended and satisfaction rate—exercise arm 93%, socialization arm 67% (significantly different across groups). Adverse events: 43 exercise-related, 23 (54%) were falls	
	Granet et al [27]	Physical: Short Physical Performance Battery, single-leg stand, gait speed, leg power (time to complete 10 chair stands), and 30-second chair stands Survey: EQ-5D (quality of life), Kessler Psychological Distress Scale (Anxiety/Depression), University of California Los Angeles Loneliness Scale-3, and Rapid Assessment of Physical Activity Usability: attendance, repeated study-specific survey utilizing Likert scales deployed after each class, and falls and adverse events		Physical: both groups improved normal gait speed, time to complete 5 chair stands, time to complete 10 chair stands, and 30-second chair stands; live group improved fast gait speed and Short Physical Performance Battery; and live>recorded in time to complete 10 chair stands and time to complete 5 chair stands. Survey: live group ↑ perceived health and motivation for physical activity; group × time interaction for physical activity habits (from Rapid Assessment of Physical Activity) with live group ↑ and recorded group ↓. Usability: dropout rate: 16% in live and 46% in recorded; attendance—89% live and 81% recorded; and similar satisfaction, Rating of Perceived Exertion, and perceived difficulty across classes. Adverse events: no falls in either group.	
	Langeard et al [28]	Physical: hand grip, leg strength (isometric knee flexion and extension), lower limb power, isometric back extension, maximal exercise test (maximal power and oxygen uptake), and body composition (fat mass and fat % using bioelectrical impedance analysis) Survey: none Usability: attendance		Physical: face-to-face>control for back extension, knee extension, and lower limb power; face-to-face=digital for changes in fat loss, maximal power, maximal oxygen uptake, lower limb power, and knee extension; videoconferencing training was not as effective as face-to-face training for improving handgrip, trunk extension, and knee flexion isometric strength. Survey: not applicable. Usability: 2 dropped from the digital group; Otherwise 100% exercise attendance for both face-to-face and digital. Adverse events: not reported.	
	Li et al [29]	Usability: study-specific satisfaction survey, retention (defined as not withdrawing from the study), and adverse events		Usability: satisfaction (n=22 from enhanced Tai Ji Quan): 82% extremely and 18% somewhat satisfied with the program, 77% found the exercise program safe, 59% found the intensity appropriate, and 55% found the exercises challenging but enjoyable. Retention: 94% Adverse events: 3 in enhanced Tai Ji Quan, 4 in standard Tai Ji Quan, and 4 in control.	
	Yerlikaya et al [30]	Physical: Berg Functional Balance Scale, timed up and go, and Postural Sway (measured with Sway Balance Mobile Application in 5 postures) Survey: WHOQOL-OLDb Usability: not applicable		Group × time interaction: not relevant for all measures. Physical: comparison pre-post within groups—the intervention group had improvements in sway, Berg Functional Balance Scale, and timed up and go, and the control group showed improvements in Berg Functional Balance Scale and timed up and go. Survey: WHOQOL-OLD improved in all groups. Adverse events: not reported.	
	Li et al [31]	Physical: 30-second chair stands, timed up and go—single task, timed up and go—dual task, and 4-Stage Balance test Usability: attendance, attrition, and adverse events		Physical: group × time interaction: Tai Ji Quan>stretch for 30-second chair stands, timed up and go single task, timed up and go dual task, and 4-stage balance test. Usability: attendance— Tai Ji Quan 80% and stretch 79%; mean number of completed sessions—Tai Ji Quan 38 (SD 9.3), stretch 37.8 (SD 7.8); overall attrition 13%. Adverse events: incidence of falls (self-reported)—15 in Tai Ji Quan and 26 in stretch; number of injurious falls—2 in Tai Ji Quan and 3 in stretch; intervention-related major adverse events 0.	
	Tomita et al [32]	Physical: gait speed/quality (width/length), hip (flexion/extension/abduction/adduction), knee (flexion/extension), and ankle (dorsi/plantar flexion) isometric strength; knee flexion and extension endurance; and Functional Independence Measure (Activity of Daily Living) Survey: Center for Epidemiological Studies—Depression, and Activities Specific Balance Confidence Usability: open-ended questions, attrition, and adverse events—falls		Physical: group × time interaction with intervention>control for balance confidence and ankle plantarflexion; post>pre for intervention for balance confidence, knee flexion and extension strength, ankle dorsiflexion and plantarflexion strength, hip adduction strength, knee flexion and extension endurance, step width, and Functional Independence Measure—motor subscale. Survey: not given. Usability: participants reported that they felt safe, encouraged, and that the exercises were easy to follow; zero attrition in exercise and 16% in control; 84.4% attendance. Adverse events: 8 falls in the intervention group and 16 in the control group.	
**Interventional nonrandomized trials**					
	Canton-Martínez et al [33]	Physical: gait speed and Senior Fitness Test Survey: Connor Davidson Resilience Scale, Hamilton Depression Rating Scale, the Geriatric Depression Scale, and the Connor-Davidson Resilience Scale Usability: adherence		Physical: exercise groups improved; significant interactions were found in lower-body strength, timed up and go, 6-meter walk, and 4-meter gait speed test. Survey: significant interactions were found in scores of the Geriatric Depression Scale, the Hamilton Depression Scale, and the resilience scores. Usability: 10 of the 20 intervention participants dropped. Adverse events: not reported.	
	Pitre et al [34]	Physical: 6-minute walk test (in-person only), 2-minute step test (remote only), 30-second chair stands, and single-leg stand Survey: 21-item Depression, Anxiety and Stress Scale and 36-item Short Form (quality of life) Usability: Functional Assessment of Comfort Employing Technology Scale and attrition		Physical: post>pre for both the intervention and control groups. Survey: 21-item Depression, Anxiety and Stress Scale—post>pre for control and post=pre for intervention; 36-item Short Form—post>pre for control and post=pre for intervention for emotional, social functioning, general health, and energy subscales. Usability: Comfort Employing Technology Scale—post>pre for control and post=pre for intervention in total and home domain subscale; attrition: 39 drops (28 intervention, 11 control). Adverse events: not reported.	
	Aksay [35]	Physical: Senior Fitness Test included 30-second chair stands, 30-second bicep curl test, 6-minute walk test, chair sit and reach, back scratch test, and 2.4-m up and go test		Physical: improvements for all measures in both sexes in the experimental group; men got worse at 30-second chair stands and improved in the 6-minute walk test and 2.4-m up and go in the control group; women improved in arm curl, 2.4-m up and go, and back scratch in the control group; the intervention group postintervention improved compared with the control group in 30-second chair stands, arm curl, 6-minute walk test, and female back scratch. Survey: not reported. Usability: not reported. Adverse events: not reported.	
	Buckinx et al [36]	Physical: gait speed (4 m) and BMI Survey: 12-item Short Form (perceived health), University of California Los Angeles Loneliness Scale-3 (loneliness), Modified Telephone Interview for Cognitive Status (cognition), and Falls Efficacy Scale-International (fear of falling) Usability: self-reported satisfaction and perceived difficulty (study-specific; Likert scale single question asked 6×), adherence, and qualitative interviews		Physical: no posttesting completed Survey: no posttesting completed No formal statistics; satisfaction—4 (36%) good and 7 (64%) excellent in the intervention group; 1 (3%) fair, 19 (59%) good, and 13 (38%) excellent in the control group; adherence—82.5% the intervention group and 85.8% in the control group; drops—2 in the intervention group and 5 in the control group; perceived difficulty: 9 (90%) moderate and 1 (10%) low in the intervention group, 20 (69%) moderate and 9 (31%) low in the control group. Adverse events: not reported.	
	VanRavenstein et al [37]	Physical: timed up and go, Berg Functional Balance Scale, 30-second chair stands, 2-minute walk, and daily steps Survey: Standardized Self-Efficacy for Exercise Scale-13 and Lubben Social Network Scale-18 (social connectedness) Usability: qualitative interviews (familiarity with technology and willingness to participate in exercise) and adherence (undefined “completion” of program)		No statistical data provided. Physical: improvement in all physical measures in both groups. Survey: control postintervention improved self-efficacy, intervention postintervention improved social network scale. Usability: interviews indicate that the group felt connected and that individualized recruitment worked well, with 100% adherence for both exercise groups. Adverse events: not reported	
**Interventional single-arm trials**					
	Lim et al [38]	Physical: Community Healthy Activities Model Program for Seniors and Modified Barthel Index Survey: EuroQoL Scale/Index, Program of Research on Integration of Services for the Maintenance of Autonomy-Frailty, and Strength, Assistance with walking, Rise from a chair, Climb stairs, and Falls Scale Usability: attendance, qualitative interviews with both participants and instructors, and injury/adverse events		Survey: no changes in quality of life, frailty, or sarcopenia risk. Usability: median attendance 54.17%, interviews indicated the intervention was considered easy by participants, classes reduced feelings of isolation, and volunteers enjoyed teaching and demonstrated competency based on fidelity checks. Adverse events: 2	
	da Silva et al [39]	Physical: Senior Fitness Test Survey: Falls Efficacy Scale—Brazil (falls) and Medical Outcome Study 36-item Short Form (quality of life) Usability: adherence and adverse events		Physical: Senior Fitness Test (subscales): ↑ upper limb strength, balance, and agility Survey: Medical Outcome Study 36-item Short Form (subscales): ↑ physical functioning, role-physical, ↓ role-emotional Usability: 20/89 participants completed the training with more than 70% frequency. Absence of identification of adverse effects	
	Kirwan et al [40]	Physical: assessments completed digitally: 30-second chair stands, 30-second arm curl, single-leg stand, sit-and-reach, 2-minute step test, and waist circumference Survey: Diabetes Self-Efficacy Scale and Patient Activation Measure (confidence in disease self-management) Usability: adherence		Physical: significant ↑ for all measures Survey: improved Diabetes Self-Efficacy Scale postintervention Usability: 70% attended 14 of 16 sessions Adverse events: not reported	
	Lee et al [41]	Usability: study-specific satisfaction survey, adherence (completed program), and specific criteria not given		Usability: 96.8% indicated the Zoom platform was easy to use, 96.8% indicated the class met expectations, 96.8% indicated there was enough time to ask questions, 93.5% felt they had learned Tai Chi, 90.3% indicated they were happy with the quality of instruction, 87.1% would recommend the class to others, and 38.7% felt a sense of community with other class members. Adherence: 73.5% completed the program. Adverse events: not reported.	
	Bagkur et al [42]	Physical: physical activity levels and sleep parameters were measured via SenseWear Pro3 Armband Survey: WHOQOL-OLD (quality of life), Pittsburgh Sleep Quality Index, and Epworth Sleepiness Scale Usability: not applicable		Physical: ↑ physical activity levels, improvements in sleep duration, sleep latency, and daytime sleepiness. Survey: improved WHOQOL-OLD, Pittsburgh Sleep Quality Index, and Epworth Sleepiness Scale Adverse events: not reported	
	Schwartz et al [43]	Survey: study specific satisfaction survey using Likert scales Usability: attendance, adherence, and adverse events		Survey: 97% would participate again, median score of satisfaction >6 on a 1-7 Likert scale. Usability: 90% attendance and 90.3% completers. Adverse events: none.	
	Wu and Keyes [44]	Physical: single-leg stand, timed up and go, sway—normal, and sway—narrow Survey: Medical Outcome Study 36-item Short Form (quality of life) Usability: attendance and adherence		Physical fitness: improved single-leg stand, timed up and go, sway—normal, and sway—narrow. Survey: Medical Outcome Study 36-item Short Form, improved social functioning subscale. Usability: attendance 78%, adherence 82.4% (3 dropped). Adverse events: not reported.	

^a^Only significant results are reported.

^b^WHOQOL-OLD: World Health Organization Quality of Life—Older Adults module.

Self-reported health status and fall risk varied widely across studies: 15 (65%) studies involved presumptively healthy older adults without known or reported comorbidities. The remaining studies (8/23, 35%) included participants with various health concerns, such as controlled mild to moderate hypertension and type 2 diabetes mellitus. Although the fall-risk category was not an explicit inclusion or exclusion criterion, the requirement that participants be ambulatory without assistive devices resulted in most samples being classified as low to moderate risk.

Among the 18 (78%) studies that reported class size, the number of participants per class ranged from as few as 4 to as many as 32. Most commonly (10/18, 56%), classes had 8 or fewer participants, although 3 (17%) studies reported class sizes greater than 15. The interventions lasted between 6 and 26 weeks. Of the studies that reported class frequency (n=20), the plurality (n=9, 45%) offered 2 sessions per week; others reported either 1 or 3 sessions per week, or did not include specific details regarding the number or timing of classes. The majority of studies (18/23) provided details on class length. Of these, 16 (89%) held sessions lasting between 30 and 60 minutes, 1 study (6%) held sessions shorter than 30 minutes, and another (n=1, 6%) held sessions longer than 60 minutes.

The level of instructor expertise was reported in some, but not all, of the reviewed studies. Specifically, 12 of the 23 (52%) studies indicated that classes were taught by a licensed or highly experienced (>10 years) exercise professional. These included 6 licensed physical therapists, 3 certified trainers, 2 Tai Chi–trained or experienced instructors, 1 kinesiologist, and 1 adapted physical educator. The remaining studies indicated that “trained” staff were used, but did not specify the level or duration of training; in others, this information was not provided. Details about the specific strategies and instructional methods employed were also lacking in most of the reviewed studies. Nevertheless, the majority of interventions (17/23, 74%) appeared to be led by a single instructor. Among those that reported using multiple instructors, 3 out of 6 (50%) employed 2 licensed instructors (eg, physical therapist or certified trainer), while the other 3 (50%) used a licensed instructor in combination with “trained volunteers.”

Outcome measures varied, but most aimed to assess aspects of physical function, particularly functional strength and dynamic balance. In the few studies that did not include these measures, the primary outcomes were acceptability or usability of a digital platform by older adults, although the training still targeted improvements in movement and physical performance. Muscular strength or endurance was the most commonly assessed metric (16 of 23 studies), with the “30-second chair stand” and “timed up and go” tests being the most frequently used in 7 and 5 studies, respectively. Other moderately common physical outcome measures were static standing balance (n=6), aerobic capacity (n=5), body composition (n=5), and gait speed (n=3). Although the magnitude of the results varied, most studies reported improvements in at least one—but often not all—of the assessed physical function metrics.

Acceptability and usability were also commonly reported, with 17 out of 23 (74%) studies including at least 1 related metric. These measures included attrition (10 of 17), adherence to prescribed activity (n=8), and attendance (n=8), with some studies reporting more than 1. Most found high acceptability, with over 80% adherence and attendance in 63% of the studies reporting each metric (5/8 for both). Among those reporting attrition (n=10), 3 (30%) studies had rates over 25%, while 5 (50%) reported rates less than 10%. Additionally, of the 23 studies, 4 (17%) used study-specific acceptability surveys, 3 (13%) conducted qualitative interviews, and 1 (4%) assessed perceived difficulty at multiple time points. These usability and qualitative data suggest that participants generally perceived the digital platforms as user-friendly and the exercises as beneficial.

Injuries specifically related to the intervention or measurement, as well as adverse events more broadly, were inconsistently reported, with only 10 studies providing any relevant data. Of these, 5 tracked all adverse events, while the remainder tracked only falls (n=4) or serious exercise-related events (n=1). Among the reporting studies, 4 indicated no adverse events, 3 reported events in both the intervention and control groups, 2 reported only intervention- or measurement-related events, and 1 reported events without specifying whether they were related to the intervention or measurement (though they were likely related). Of the 6 studies reporting adverse events, 4 provided severity details, with only 1 reporting any serious adverse events (injurious falls).

Additional quality of life data were collected via survey in 15 of the 23 studies. Validated quality of life instruments were most common (10 of 15), although the specific tools varied widely, with no single survey used in more than 2 studies. Depression was also relatively frequently assessed (6 studies). Other occasionally measured outcomes included self-reported physical activity (n=4 studies), fear of falling (n=4 studies), resilience (n=3 studies), self-efficacy to exercise (n=2 studies), and comfort with technology (n=1 study). The vast majority of studies reported improvements in quality of life, resilience, and self-efficacy, as well as reductions in depression following the digital intervention.

Further details regarding the study populations, interventions, and significant results are provided in Tables 1 and 2. Table 1 summarizes the sample and intervention characteristics of the reviewed manuscripts, while Table 2 outlines the measures used and the results reported.

## Discussion

### Principal Findings

This scoping review examined the quantity and quality of publications on digitally delivered, instructor-led group exercise classes aimed at improving physical function and preventing falls among older adults. Eligible studies included randomized and nonrandomized trials, as well as single-arm pilot studies, conducted over at least 6 weeks, regardless of the number of weekly sessions.

Overall, only a small number of studies met our inclusion criteria (n=23). Considering the wide range of exercise programs now delivered online, the growing internet use among older adults, and the increasing public health emphasis on the benefits of exercise for this population, the limited number of eligible studies was somewhat surprising. We anticipate that the number of eligible studies will grow substantially as more exercise programs are designed and delivered digitally. Of the articles published between 2006 and July 2024 that met our inclusion criteria, 11 were randomized controlled trials, while the remainder comprised 7 single-group studies and 5 two-group pre/postcomparison nonrandomized studies. The studies represented populations from 14 countries, offering noteworthy racial, ethnic, and geographical diversity among older adults.

Most studies included in this review did not compare digital programs with in-person equivalents, making it impossible to determine whether digital delivery is superior—or even noninferior—to traditional “live” exercise programming. Nevertheless, digitally delivered programs generally improved physical outcomes related to lower limb strength and balance, with some also demonstrating gains in cardiovascular function and upper body strength. In addition, evidence suggests these interventions tended to enhance overall quality of life and reduce depression among participants.

It is also worth noting that participants responded positively to the digitally delivered experience when queried. Although rarely reported [32,34,36,37], studies that assessed older adults’ comfort with technology found that those who took part in the interventions improved their ability to use technology in general, and videoconferencing in particular. More importantly, the relatively high adherence and attendance rates across these studies suggest that technological challenges did not substantially reduce participation. In fact, adherence rates were comparable to those seen (~70%) in healthy older adults [45] and the 60%-70% observed in clinically defined groups [46] engaged in live exercise interventions. Although this level of adherence to a technological intervention is promising, it is important to note that individuals willing to participate in such interventions may already be more knowledgeable about, or more comfortable with, technology than the general population. Given previous findings on difficulties with technology among some older adults [5,6], caution should be exercised when generalizing these results to other older adult populations.

Although many of the included studies did not provide detailed information on injuries, it appeared that few adverse events—and very few serious adverse events—resulted from digitally delivered exercise programs. High-quality evidence on injury rates specifically among older adults initiating an unsupervised, video-based exercise program is lacking. However, during the COVID-19 lockdown, a survey of British citizens found that approximately 14% of the adult population sustained an injury while exercising, with more than 30% of these injuries occurring during video- or app-led exercise [47]. While some of these injuries were likely due to overuse or unavoidable factors, it is reasonable to assume that real-time digital classes led by a live instructor may help address certain safety concerns associated with prerecorded, video-based instruction. In particular, monitoring proper form and progression—especially when paired with personalized instruction and feedback—can reduce injuries related to limited exercise knowledge or fatigue-induced changes in movement. However, even in live digital formats, instructors may need to position themselves at a considerable distance from their recording device(s), which often also serve as their class display device (eg, laptop or tablet). As such, viewing participants with enough clarity to offer meaningful, form-based feedback can be challenging. In larger classes, participants may appear in increasingly smaller windows, or the instructor may have to forgo viewing all participants simultaneously—both of which hinder effective observation. These limitations make it difficult for a digital instructor to provide timely and precise feedback. While this review does not aim to draw a definitive conclusion on the matter, it is plausible that having a second instructor dedicated solely to real-time feedback could enhance safety and sustain participant engagement.

The limited reporting regarding the specifics of the intervention—such as instructor expertise, exercise details and progressions, at-home exercise prescriptions (if any), and, perhaps most importantly, the amount and type of instructor feedback—is worth highlighting. Although these details could likely be obtained by directly contacting the study authors, their absence in the published reports substantially limits the ability to compare interventions and assess reproducibility. Given that “exercise” can vary widely in domain (eg, cardiovascular, strength), intensity, and level of instruction, explicitly reporting these details is essential. While this holds true for exercise interventions in general, it is particularly critical for emerging digitally delivered programs—especially those targeting geriatric populations and other groups at higher risk for injury. Similarly, the limited reporting of adverse events hinders the ability to assess the relative safety of these interventions and reduces the confidence with which they can be recommended to at-risk individuals.

### Strengths and Limitations

A strength of our study is the diversity of the sample included in the review, encompassing 14 countries with multiracial, multiethnic, and economically varied populations worldwide. This breadth—though an incidental outcome—underscores the global interest in fall prevention, particularly through exercise-based interventions. Another strength lies in the variety of outcome measures used in most, though not all, of the studies reviewed, offering a more comprehensive understanding of intervention effects.

Although this scoping review offers a reasonably comprehensive overview of currently available digital exercise programs, several limitations should be acknowledged. First, our conclusions are based on the evidence presented in the reviewed articles and on the assumption that, had there been meaningful injuries (in either number or severity), they would have been reported. However, because most of the reviewed studies did not include adverse event data, our conclusions regarding safety may be unreliable. Perhaps the most significant limitation of this review is the absence of any formal assessment of the quality of the included studies and their reported data. While this egalitarian approach ensures the inclusion of a wide range of studies, it does not account for variations in methodological rigor, the presence of a control group, or sample size. Furthermore, no meta-analysis was conducted to determine the actual effect size of this type of intervention. Consequently, although general conclusions can be drawn, caution is warranted when interpreting the overall efficacy of digital exercise interventions for improving physical function, enhancing quality of life, or reducing fall risk. Additionally, because our review focused exclusively on digital interventions in (mostly) healthy individuals, these findings are not generalizable to clinically relevant subpopulations who may stand to benefit most from high-quality digital programs. Finally, although the 3 databases used in our search likely captured all relevant manuscripts published in English, it is possible that including additional databases could have yielded further relevant studies.

### Insights/Future Directions

The results of this scoping review indicate that digitally delivered, instructor-led exercise interventions for older adults show considerable promise, offering improved accessibility while fostering participant engagement and emphasizing safety. Despite heterogeneity in design, the reviewed studies collectively suggest that such interventions can support meaningful improvements in lower-body strength, balance, and quality of life, while maintaining high acceptability and adherence. This suggests a conceptual framework in which digital group exercise programs enhance physical function through both physiological and psychosocial pathways—leveraging not only movement but also social connectedness, routine, and perceived competence with technology. The presence of a live instructor appears central to this process, acting as both a facilitator of physical safety and a source of motivation and encouragement. Yet, variability in reporting across studies—particularly in instructional strategies, feedback mechanisms, exercise progressions, and the number and severity of adverse events—underscores the need for standardization. This is especially critical when addressing safety and adverse event reporting. Studies should explicitly state when no injuries or adverse events occurred, rather than omitting safety information from reports on intervention effects. Comprehensive tracking and reporting of adverse events, including those occurring outside of direct instruction, would not only help establish the relative safety of such interventions but also clarify which populations are most appropriate for participation. Similarly, detailed reporting of exercise programming, progression, and instructor training and engagement strategies is essential to enable replication, synthesis, and iterative development. As digital instruction continues to proliferate and evolve, situating these interventions within broader determinants of healthy aging—such as autonomy, self-efficacy, and digital literacy—can inform both future research and practical implementation.

## Appendix

Multimedia Appendix 1 Search terms by public database used for identifying potential studies for review.

Multimedia Appendix 2 PRISMA-ScR (Preferred Reporting Items for Systematic Reviews and Meta-Analyses extension for Scoping Reviews) checklist.

Multimedia Appendix 3 Headers used for data extraction.

## Abbreviations

CENTRAL: Cochrane Controlled Register of Trials
PRISMA-ScR: Preferred Reporting Items for Systematic reviews and Meta-Analyses extension for Scoping Reviews

## Data Availability

Lists of considered studies by database and data extraction grids are available upon reasonable request from the corresponding author.
