# Supplementary material for: Digitally Delivered, Group-Based Exercise Interventions for Older Adults: Scoping Review
Source: J Med Internet Res. 2025 Sep 3;27:e73578. doi: 10.2196/73578 (PMC12444216; doi:10.2196/73578)
Supplement: Multimedia Appendix 1 [file jmir_v27i1e73578_app1.docx]

**Supplementary Table: Search Terms by Public DataBase used for Identifying Potential Studies for Review**

MEDLINE via Pubmed

| #1 | "Aged"[Mesh] OR "Elderly" OR "Frail Elderly" OR "Elderly, Frail" OR "Frail Elders" OR "Elders, Frail" OR "Frail Elder" OR "Functionally-Impaired Elderly" OR "Elderly, Functionally-Impaired" OR "Functionally Impaired Elderly" OR "Frail Older Adults" OR "Adults, Frail Older" OR "Frail Older Adult" OR "Older Adult, Frail" OR "Older Adults, Frail" OR "Elderly" OR "Older Adult" OR "Older Adults" OR "Postmenopause"[Mesh] OR "Postmenopausal Period" OR "Period, Postmenopausal" OR "Post-Menopause" OR "Post Menopause" OR "Post-Menopause" OR "Post-menopausal Period" OR "Period, Post-menopause" OR "Post menopausal Period" |
| --- | --- |
| #2 | "Exercise"[Mesh] OR "Exercises" OR "Physical Activity" OR "Activities, Physical" OR "Activity, Physical" OR "Physical Activities" OR "Exercise, Physical" OR "Exercises, Physical" OR "Physical Exercise" OR "Physical Exercises" OR "Acute Exercise" OR "Acute Exercises" OR "Exercise, Acute" OR "Exercises, Acute" OR "Exercise, Isometric" OR "Exercises, Isometric" OR "Isometric Exercises" OR "Isometric Exercise" OR "Exercise, Aerobic" OR "Aerobic Exercise" OR "Aerobic Exercises" OR  "Exercises, Aerobic" OR "Exercise Training" OR "Exercise Trainings" OR "Training, Exercise" OR "Exercise Movement Techniques"[Mesh] OR "Exercise Movement Technics" OR "Pilates-Based Exercises" OR "Exercises, Pilates-Based" OR "Pilates Based Exercises" OR "Pilates Training" OR "Training, Pilates" OR "Exercise Therapy"[Mesh] OR "Remedial Exercise" OR "Exercise, Remedial" OR "Remedial Exercises" OR "Therapy, Exercise" OR "Exercise Therapies" OR "Therapies, Exercise" OR "Rehabilitation Exercise" OR "Exercise, Rehabilitation" OR "Exercises, Rehabilitation" OR "Rehabilitation Exercises" OR "Motor Activity"[Mesh] OR "Activities, Motor" OR "Activity, Motor" OR "Motor Activities" OR "Resistance Training"[Mesh] OR "Training, Resistance" OR "Strength Training" OR "Training, Strength" OR "Weight-Lifting Exercise Program" OR "Weight Lifting Exercise Program" OR "Weight-Lifting Exercise Programs" OR "Weight-Bearing Strengthening Program" OR "Strengthening Programs, Weight-Bearing" OR "Weight Bearing Strengthening Program" OR "Weight-Bearing Strengthening Programs" OR "Weight-Bearing Exercise Program" OR "Exercise Programs, Weight-Bearing" OR "Weight Bearing Exercise Program" OR "Weight-Bearing Exercise Programs" OR "Postural Balance"[Mesh] OR "Posture Equilibrium" OR "Equilibrium, Posture" OR "Balance, Postural" OR "Postural Equilibrium" OR "Equilibrium, Postural" OR "Posture Balance" OR "Balance, Posture" OR "Musculoskeletal Equilibrium" OR "Equilibrium, Musculoskeletal" OR "Postural Control" OR "Control, Postural" OR "Postural Controls" OR "Posture Control" OR "Control, Posture" OR "Posture Controls" OR "Circuit-Based Exercise"[Mesh] OR "Circuit Based Exercise" OR "Circuit-Based Exercises" OR "Circuit Training" OR "Training, Circuit" OR "Agility" OR "Agility Exercise" OR "Agility Exercises" OR "Agility Training" OR "Agility Program" OR "Range of Motion, Articular"[Mesh] OR "Joint Range of Motion" OR "Joint Flexibility" OR "Flexibility, Joint" OR "Range of Motion" OR "Passive Range of Motion" OR "Flexibility Exercise" OR "Flexibility Exercises" OR "Flexibility Training" OR "Flexibility Program" |
| #3 | "Digital Technology"[Mesh] OR "Digital Technologies" OR "Technologies, Digital" OR "Technology, Digital" OR "Digital Electronics" OR "Electronics, Digital" OR "Internet-Based Intervention"[Mesh] OR "Internet Based Intervention" OR "Internet-Based Interventions" OR "Intervention, Internet-Based" OR "Interventions, Internet-Based" OR "Web-based Intervention" OR "Intervention, Web-based" OR "Interventions, Web-based" OR "Web based Intervention" OR "Web-based Interventions" OR "Online Intervention" OR "Intervention, Online" OR "Interventions, Online" OR "Online Interventions" OR "Internet Intervention" OR "Internet Interventions" OR "Intervention, Internet" OR "Interventions, Internet" OR "Telerehabilitation"[Mesh] OR "Tele-rehabilitation" OR "Tele rehabilitation" OR "Remote Rehabilitation" OR "Rehabilitation, Remote" OR "Virtual Rehabilitation" OR "Rehabilitation, Virtual" OR "Virtual Rehabilitations" OR "Virtual" OR "Virtual classes" OR "Telemedicine"[Mesh] OR "Tele-Referral" OR "Tele Referral" OR "Tele-Referrals" OR "Virtual Medicine" OR "Medicine, Virtual" OR "Tele-Intensive Care" OR "Tele Intensive Care" OR "Tele-ICU" OR "Tele ICU" OR "Mobile Health" OR "Health, Mobile" OR "mHealth" OR "Telehealth" OR "eHealth" |
| #4 | #1 AND #2 AND #3 |

Cochrane Center Register of Controlled Trials (CENTRAL)

| #1 | "Aged" |
| --- | --- |
| #2 | "Frail Elderly" |
| #3 | "Postmenopause" |
| #4 | #1 OR #2 OR #3 |
| #5 | "Exercise" |
| #6 | "Exercise Movement Techniques" |
| #7 | "Exercise Therapy" |
| #8 | "Motor Activity" |
| #9 | "Resistance Training" |
| #10 | "Postural Balance" |
| #11 | "Circuit-Based Exercise" |
| #12 | "Range of Motion, Articular" |
| #13 | #5 OR #6 OR #7 OR #8 OR #9 OR #10 OR #11 OR #12 |
| #14 | "Internet-Based Intervention" |
| #15 | "Telerehabilitation" |
| #16 | "Telemedicine" |
| #17 | #14 OR #15 OR #16 |
| #18 | #4 AND #13 AND #17 |

EMBASE

| #1 | 'aged'/exp OR 'frail elderly'/exp OR 'postmenopause'/exp |
| --- | --- |
| #2 | 'exercise'/exp OR 'kinesiotherapy'/exp OR 'motor activity'/exp OR 'resistance training'/exp OR 'body equilibrium'/exp OR 'circuit training'/exp OR 'range of motion'/exp |
| #3 | 'web-based intervention'/exp OR 'telerehabilitation'/exp OR 'telehealth'/exp OR 'telemedicine'/exp |
| #4 | #1 AND #2 AND #3 |
