# Supplementary material for: Digitally Delivered, Group-Based Exercise Interventions for Older Adults: Scoping Review
Source: J Med Internet Res. 2025 Sep 3;27:e73578. doi: 10.2196/73578 (PMC12444216; doi:10.2196/73578)
Supplement: Multimedia Appendix 3 [file jmir_v27i1e73578_app3.pdf]

Study Identification/design				
Title	Study ID<br>(1st author last name and yr of publication)	Country	Study design	Study aim

Sample characteristics (if more than one group, report per arm)					
Sample characteristics (if more than one group, report per arm)	Sex	Race/ethnicity	Age	Comorbidities/health conditions?	Mobility e.g., any information on being ambulant, needing assistive device)

Intervention					
Total duration	Frequency	Characteristics (such as A:aerobic, S:strength, B:balance;F:flexibility)	Platform/method of delivery	Instructor (description/training)	Group or individual?

	Control/Comparison				
	Yes or No?	Description of control/comparison	Measures of physical function and muscular strength (such as validated measures of gait speed, chair-sit-to-stand), TUG, grip strength, balance, posture)	Which ones?	Results (per arm)

Outcomes of interest - Primary					
Adverse events (such as falls, injurious falls, fractures, other exercise induced injuries, e.g., strains and sprains, resulting in medical intervention and missed days of exercise)	Related to the intervention?	Results (per arm)	Quality of life (using validated questionnaires)	Which ones?	Results (per arm)

Outcomes of interest - Secondary					
Measures of physical fitness (e.g., CRF, flexibility, balance, strength)	Which ones?	Results (per arm)	Satisfaction (overall rating of satisfaction with the intervention, or as described by the primary authod)	Results (per arm)	Adherence to the intervetion (attendance to exercise sessions, or other measures as reported by primary author)

	Key conclusions
Results	
